# History of the Case of a Very Extensive Burn —Successfully Treated According to Mr. Kentısh's Method

**Published:** 1807-10

**Authors:** 

**Affiliations:** Philadelphia


### *History of the Case of a very extensive Burn—successfully treated according to Mr. Kentish's method.* By Dr. Coxe, of Philadelphia.

THE following case is so strongly in favour of Mr. Kentish's plan for the treatment of burns, that I feel assured its diffusion will be productive of advantage.

Anne M'Quicker, five years of age, living with Mr. Robert Smith, No. 28, South Second-street.- On the 11th of January of the present year, her clothes, which were of thin callico, (a miserable dress for the inclement season), took fire whilst she was sitting by the stove, and were nearly burnt off her back. The child ran in great agony into the street, in the midst of the snow; and by the endeavours of some to put out the flames, the clothes were stript off her, carrying with the cinders, the cuticle and skin in some places, to an extent of surface, whose circumference, *five feet* in length of string, would barely surround. She was taken into a neighbour's house, almost in a state of nakedness, and a most miserable spectacle, arising from the mixture of blood and half-burnt tinder, &c. adhering to this extensive injured surface.

A medical gentleman, then in the neighbourhood, recommended the application of cold milk to the burn, and laudanum laudanum to be used internally. As no advantage was derived from this, and as the child suffered the most violent pain, I was requested to see her, as I happened to be at one of the neighbours. Uncertain of meeting with the gentleman who had previously prescribed for the child, and anxious, if possible, to afford her some relief, I concluded to go, and found her, even worse than had been represented. She was suffering under one of the most violent chilly fits I ever witnessed, which seemed as if it would carry her off; and the pain of the burn appeared to be absorbed in the superior suffering from the chill; for she exclaimed continually (whilst her teeth were violently chattering in her head), "cover me! cover me!"—The burn extended from the right axilla to below the great trochanter of the same side—over the right clavicle and breast, and beyond the sternum in some parts, over the abdomen beyond the linea alba and the upper parts of the pudenda—backwards it extended to the spine, nearly in a straight line down—and the greater portion of the inferior part of the arm was either raw or in blisters. The fire had extended its influence considerably beyond the boundary of the sore, as was evident from the erysipelatous blush, and from several small blisters scattered here and there.

It appeared scarcely possible that so young a child should survive so extensive a wound; and even if the first violence of the injury was overcome, it remained doubtful if the copious suppuration would not be greater than she could support. Nevertheless, I felt persuaded that nothing could save her but the plan proposed by Mr. Kentish; and I ordered immediately the spirits of turpentine on rags to the whole surface of the sore. From the hurry, I neglected to have it warmed, which would probably have aided in sooner removing the chill. In the mean time she took thirty drops of laudanum, and the rags were kept constantly wet till the liniment was prepared, as a permanent application. The laudanum was repeated, as the chill did not entirely subside, and the pain continued violent several hours. She was directed the use of wine and water to drink; and the liniment was applied anew at bed-time, with some more laudanum; which relieved her, but did not prevent a restless night.

The following day (the 12th) the pain, though great, was much alleviated, and the liniment was continued with occasional doses of laudanum. She complained of great difficulty of swallowing, which was ascribed to the flame having having been drawn into the fauces. 13th. Symptoms the same; slight appearance of suppuration; the blackness of the sore is now chiefly removed, by the tinder, &c. adhering to the ointment, and coming away with the dressing. On the 14th, suppuration commenced, and became daily more copious; she was ordered laxative medicines, and put on a strict diet. One part was conspicuous, of about five or six inches diameter, on the lower part of the abdomen, bounded by the pubes, the linea alba, the ilium, and a line from thence to the navel, which seems to be so much destroyed that a deep slough may probably come away. As suppuration was now well advanced, I applied, about the 16th, Turner's cerate, chiefly as an estimate of the value of either application in such an extensive burn. The result was, the child was worse all day and night, with additional fever and pain, and considerable alteration in the healthy aspect of the sore. The liniment consequently was re-applied, with great relief: her bowels were moved with senna and manna, and by the next day things were as favourable as before.

20th. Pain still very considerable, from the great extent of the sore, which precludes the smallest motion without rubbing some part of it. She is obliged to be lifted bodily out of bed, in order to be dressed, which causes much pain. as she is forced to stand the whole time. The suppuration became now very copious; I therefore again tried Turner's cerate, and finely levigated; but the alteration for the worse in twenty-fours was so great, that I was in no hurry to renew the trial. The turpentine liniment again restored its healthy state.

During all this time she was very strictly dieted, and so highly excitable was her system, that from foolishly giving her a small piece of a chicken, for dinner, which she thought she could eat, a violent degree of fever took place, which kept her restless and uneasy all night, with augmented pain of sore: and when dressed in the morning, in place of fine healthy granulations and suppuration, an ugly ill-conditioned ulcer, with sanious discharges, presented itself to view. Purging and close attention to diet, once more, in a short time, overcame this, and the edges were rapidly cicatrizing.

It was now thought advisable to get her into the hospital, as from the extent of sore, it was presumed it would take a great while to heal; and she was admitted January the 31st. In a short time it became necessary to prescribe the bark in decoction, with jellies, &c. Serpentaria also, and other other remedies calculated for the debilitated state of her stomach, were employed, by which she recruited; the sore healed rapidly under the use of Turner's cerate, so that by the middle of March it was not larger than the palms of both hands, and confined to that part which I have stated, as being most injured by the fire. A solution of white vitriol at this time hastened its cicatrization; and on the 11th of May she was discharged cured. Had she not accustomed herself to pick the sore, it probably would have been well two or three weeks sooner; as for four or five weeks previous to this, it had not been more than from two to three inches in diameter. On measuring the circumference of the cicatrix before she left the hospital, it required a string of *four feet six inches* to surround it. I have observed, that five would not have surrounded the extent of the injury sustained, in the first instance, especially if that of the arm is included, which in the present measurement is not adverted to.

The above instance, if I had any doubts before of the efficacy of Mr. Kentish's plan of treating burns, would have completely silenced them; and it gives me pleasure to add, that the physician who first prescribed for the patient, and who had had frequent opportunity of seeing very extensive injuries from burns and scalds, on the large plantations of some of the West-India islands, was beyond measure gratified at the powerful and rapid effects of the plan pursued above, which he said he should have been fearful of using, had he not been convinced of its mildness and utility by so striking an example.

*Philadelphia, July* 1, 1805.

